# Candida lusitaniae as an Unusual Cause of Otomycosis: A Case Report and Literature Review

**DOI:** 10.7759/cureus.115614

**Published:** 2026-09-01

**Authors:** Jalal Abderrahmani, Mohammed Amine Nejjari, Aziza Hami

**Affiliations:** 1 Laboratory of Microbiology, Mohamed VI University Hospital, Faculty of Medicine and Pharmacy, University Mohammed the First, Oujda, MAR; 2 Laboratory of Parasitology and Mycology, Central Laboratory Department, Mohamed VI University Hospital, Faculty of Medicine and Pharmacy, University Mohammed the First, Oujda, MAR

**Keywords:** amphotericin b, antifungal susceptibility, candida lusitaniae, maldi-tof mass spectrometry, non-albicans candida infection, otomycosis

## Abstract

Otomycosis is a fungal infection of the external auditory canal that may extend to the tympanic membrane. Although commonly caused by *Aspergillus* and *Candida* species, infections due to uncommon fungal pathogens may present diagnostic and therapeutic challenges. We report the case of a 46-year-old woman who presented to the Department of Otorhinolaryngology (ENT) with intense pruritus of the right external auditory canal, associated with a whitish discharge and tympanic perforation on otoscopic examination. Mycological analysis of the ear discharge identified *Candida lusitaniae*. To our knowledge, this represents the first case of otomycosis due to *C. lusitaniae* reported in our department, underscoring the importance of accurate fungal identification for guiding appropriate antifungal therapy in atypical or refractory otomycosis.

## Introduction

Otomycosis is a superficial fungal infection of the external auditory canal that accounts for a substantial proportion of cases of otitis externa worldwide, particularly in hot and humid climates [1]. It is most commonly caused by *Aspergillus *species (notably *A. niger* and *A. fumigatus*) and, less frequently, by *Candida *species, chiefly *C. albicans* [1]. Recognised risk factors include local trauma (cotton-tip swab use, hearing-aid or earplug wear), chronic moisture exposure (swimming, humid environments), prior antibiotic or corticosteroid use, immunosuppression, and diabetes mellitus [1]. While generally considered a benign and self-limiting condition, otomycosis can become recurrent or refractory, especially when the causative organism is unusual or when local risk factors persist, underscoring the need for accurate mycological identification to guide treatment [1,2].

Among the less common agents implicated in otomycosis, *Candida lusitaniae*, the anamorph of *Clavispora lusitaniae*, deserves particular attention. Originally described as a commensal of the digestive tract of warm-blooded animals, this opportunistic yeast was long considered rare and poorly pathogenic. It is now recognised as an emerging infectious agent, primarily implicated in nosocomial infections among immunocompromised patients [3]. Its clinical significance lies chiefly in its unusual antifungal susceptibility profile: it can rapidly develop acquired resistance, notably to amphotericin B and, less consistently, to fluconazole [4,5]. Biologically, *C. lusitaniae* is characterised by germ-tube negativity and the absence of true hyphae, in contrast to *C. albicans*. It also exhibits phenotypic variability, including variable fermentation and assimilation profiles for sugars such as glucose, maltose, and galactose. On microscopic examination, it may form variable pseudohyphae, which can occasionally appear branched or curved. These characteristics, together with its variable colony morphology on differential media such as CHROMagar *Candida *(CHROMagar, Paris, France), may complicate its identification and lead to confusion with other *Candida *species [3,4]. This phenotypic plasticity, combined with its ability to colonise diverse human and hospital reservoirs (digestive flora, skin, contaminated medical devices), facilitates its transition from a commensal to a pathogenic state in a range of anatomical sites in immunocompromised patients and, more rarely, in immunocompetent individuals.

Although *C. lusitaniae* has been described in various clinical settings, its involvement in otomycosis remains exceptionally rare, and the available literature on this specific localisation is limited, leaving its clinical characteristics and diagnostic challenges in ear infections insufficiently documented. This case therefore contributes to the sparse literature on *C. lusitaniae *otomycosis, expanding current knowledge of this pathogen as a cause of otitis and highlighting the importance of considering uncommon *Candida *species in the differential diagnosis of external ear fungal infections.

## Case presentation

A 46-year-old immunocompetent woman, with a known history of hepatic steatosis and chronic moderate hypoalbuminaemia, presented to the Otorhinolaryngology (ENT) Outpatient Clinic in March 2026 with intense pruritus of the right ear evolving over approximately 10 days. Otoscopic examination revealed a creamy mycotic discharge associated with partial tympanic perforation and no signs of active otitis media (Figure 1). As no prior otoscopic examination was available, it could not be determined whether this perforation pre-existed or was related to the current fungal infection. No recent local antifungal or antibiotic treatment had been administered. The case history revealed no repeated swimming, no use of topical corticosteroids, and no associated chronic ENT pathology.

**Figure 1:**
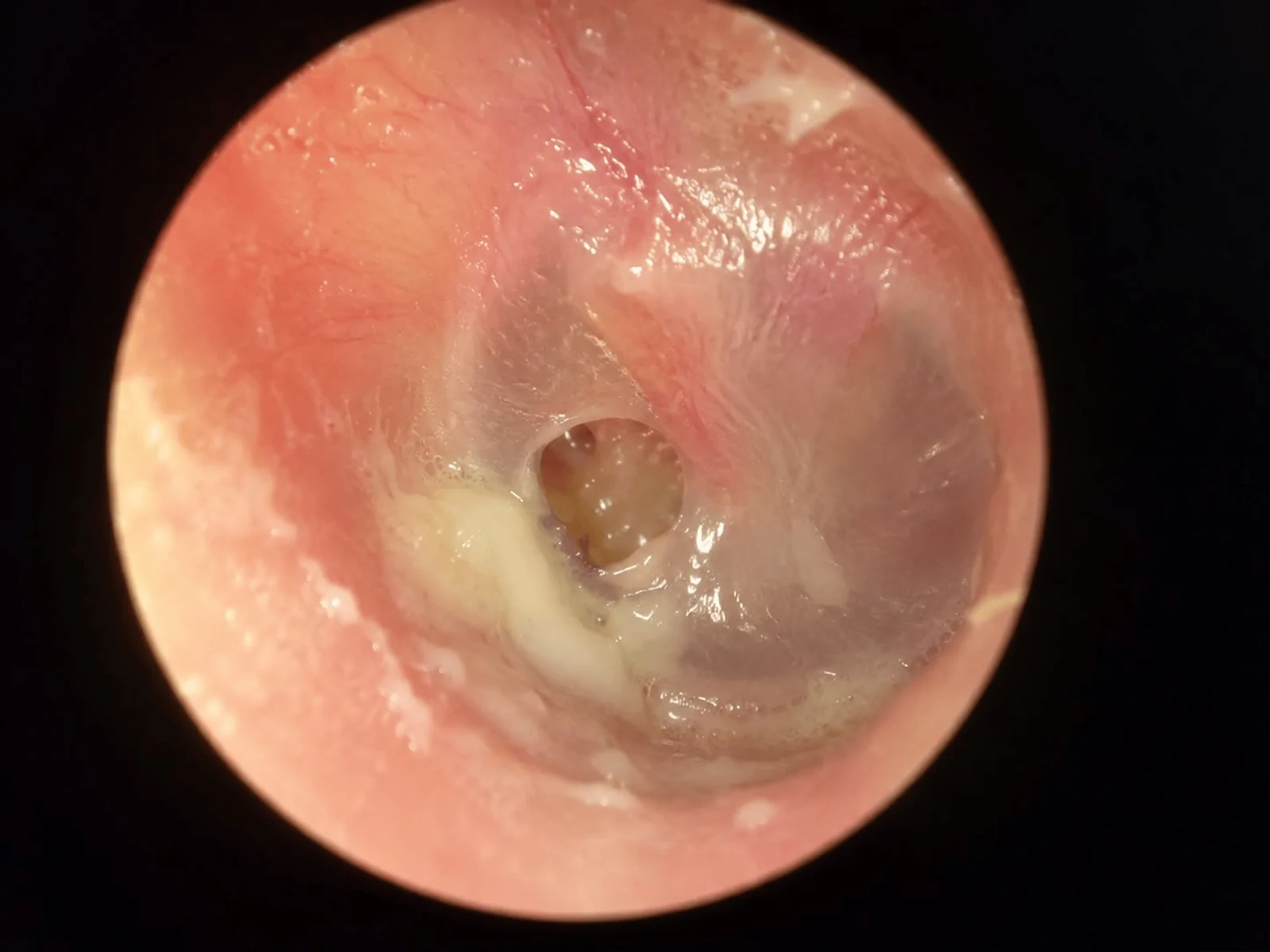
Otoscopic examination of the patient's ear canal at presentation showing whitish fungal debris and a central tympanic membrane perforation.

A sample was collected from the secretions of the external auditory canal using a sterile auricular swab and sent to the mycology laboratory. The specimen was inoculated onto Sabouraud agar and incubated at 25°C. After 48 hours of incubation, abundant growth of smooth, moist, creamy-white colonies was observed (Figure 2).

**Figure 2:**
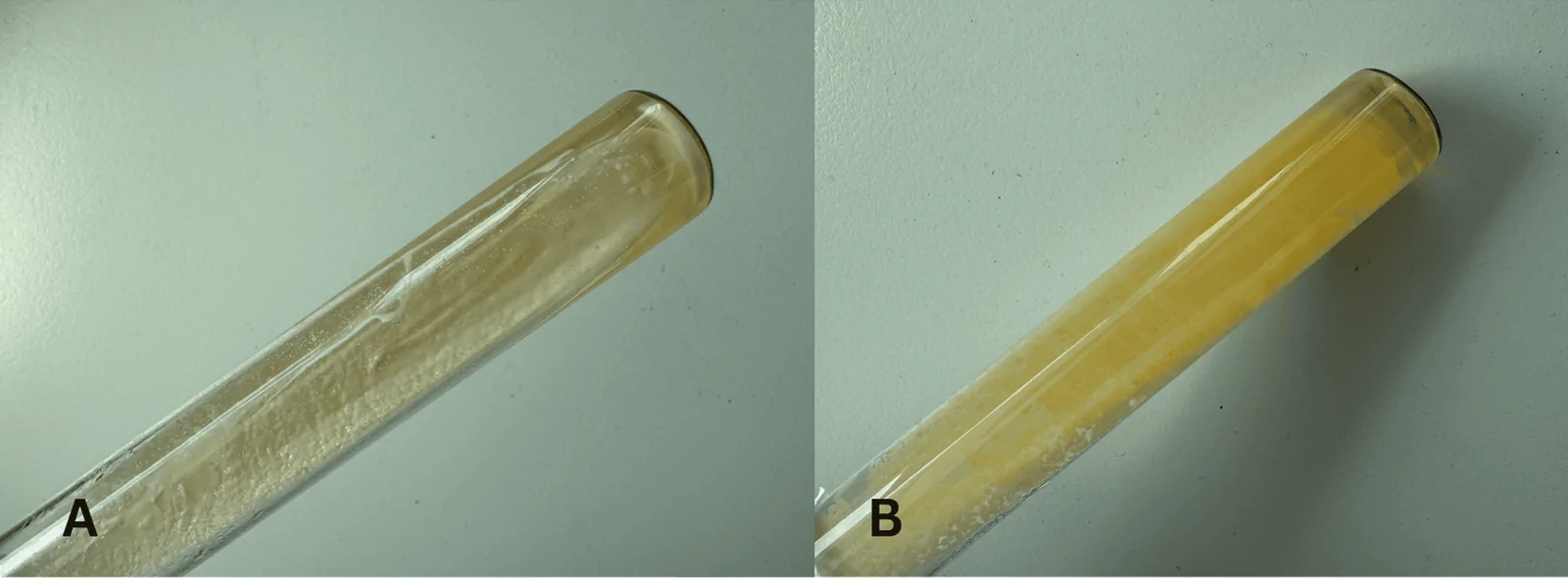
Culture on Sabouraud agar without chloramphenicol: (A) reverse side; (B) obverse side.

Direct microscopic examination of a fresh preparation from the Sabouraud agar culture showed oval to round budding yeast cells without pseudohyphae formation, consistent with a non-filamentous yeast. A subculture was then performed on CHROMagar *Candida *for diagnostic orientation. After incubation at 37°C, the colonies initially appeared whitish to cream in colour. With prolonged incubation, they progressively developed purple pigmentation, suggesting a non-albicans *Candida *species (Figure 3).

**Figure 3:**
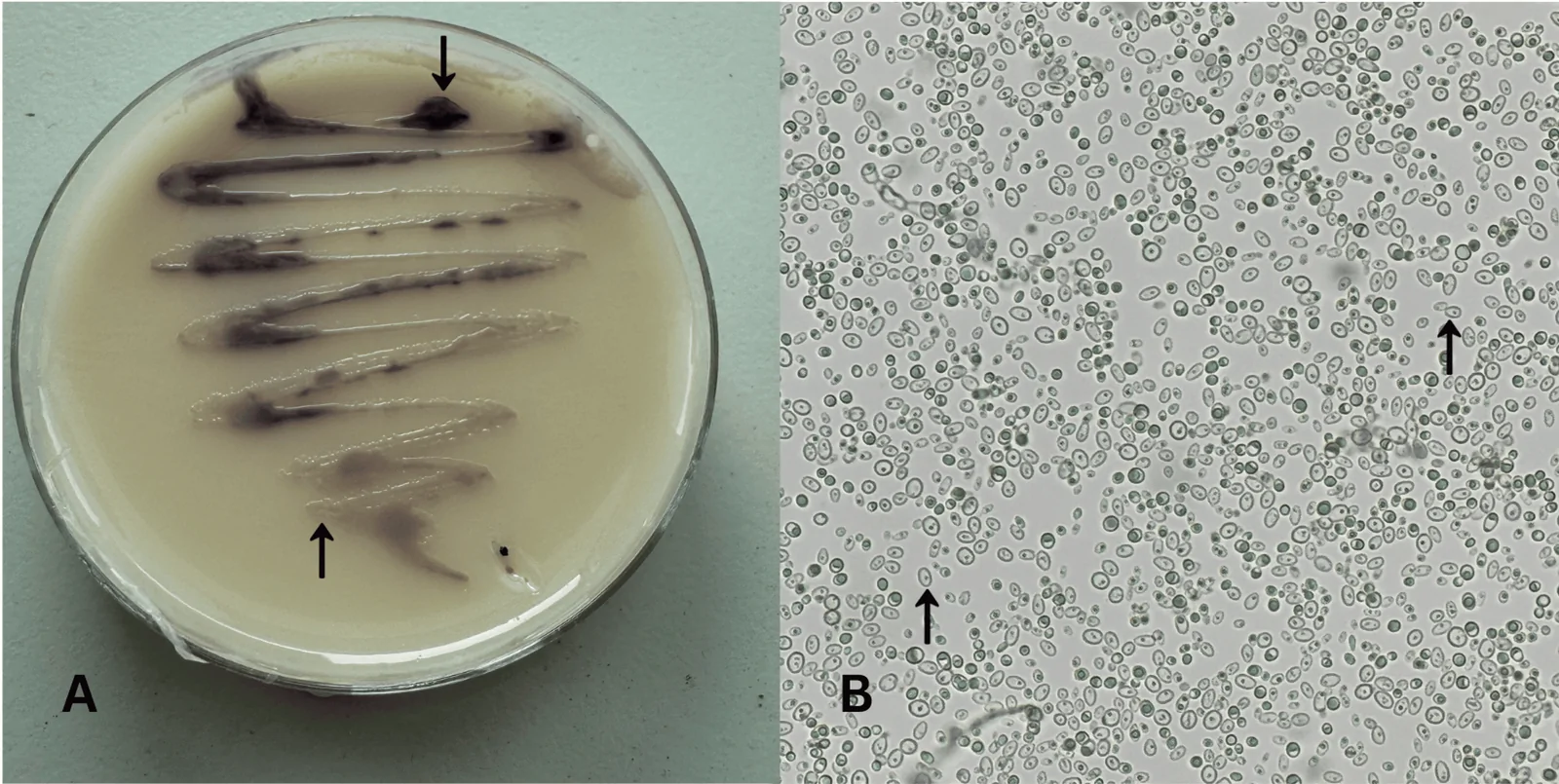
Fungal colonies on Sabouraud agar culture after prolonged incubation showing variable pigmentation ranging from whitish/cream to purple (A). Direct microscopic examination of the culture (×400 magnification) showing oval to round budding yeast cells without pseudohyphae formation (B). In (A), the arrows indicate colonies displaying the purple pigmentation that developed on the chromogenic agar after prolonged incubation. In (B), the arrows indicate the oval to round budding yeast cells observed on direct microscopic examination.

Identification was confirmed by matrix-assisted laser desorption ionisation-time of flight (MALDI-TOF) mass spectrometry (Bruker Biotyper; Bruker Daltonics, Bremen, Germany), which identified *Candida lusitaniae* (*Clavispora lusitaniae*) with a score of 2.3, with all 10 of the top spectral matches concordantly identifying the same species. According to manufacturer-recommended interpretive criteria, a score ≥ 2.00 indicates reliable species-level identification [6], confirming the diagnosis (Table 1). No antifungal susceptibility testing was performed. Topical treatment consisted of nystatin powder (100,000 units/g) insufflation into the external auditory canal three times daily for two weeks, combined with regular drying of the ear canal. The clinical course was favourable, with complete resolution of pruritus and discharge at the two-week follow-up visit, and no recurrence. At the one-month follow-up, the patient reported no symptoms and otoscopic examination showed complete resolution of the external auditory canal inflammation. No treatment-related complications were observed. Given the persistent tympanic membrane perforation, the patient was subsequently scheduled for tympanoplasty.

**Table 1:** Top 10 MALDI-TOF reference spectra matches identifying the clinical isolate as Clavispora lusitaniae (Candida lusitaniae). Score interpretation (Bruker Biotyper criteria): ≥2.00 = high-confidence species-level identification; 1.70-1.99 = secure genus-level identification, probable species-level; <1.70 = unreliable identification [6]. MVD: MALDI Validation Database (Bruker validated reference spectrum); MALDI: matrix-assisted laser desorption ionisation

Rank	Reference strain	Score value	Confidence
1	*Clavispora lusitaniae* 13043263_3 MVD	2.30	High confidence
2	Clavispora lusitaniae	2.19	High confidence
3	Clavispora lusitaniae	2.16	High confidence
4	Clavispora lusitaniae	2.07	High confidence
5	Clavispora lusitaniae	1.97	Secure genus identification
6	*Clavispora lusitaniae* 13026679_3 MVD	1.95	Secure genus identification
7	Clavispora lusitaniae	1.94	Secure genus identification
8	Clavispora lusitaniae	1.89	Secure genus identification
9	Clavispora lusitaniae	1.88	Secure genus identification
10	Clavispora lusitaniae	1.87	Secure genus identification

## Discussion

Otomycosis due to *C. lusitaniae* represents an exceptional clinical entity. While yeasts of the genus *Candida *account for approximately 10% to 15% of otomycosis cases, the majority are attributed to *C. albicans* and *C. parapsilosis*, species recognised for their cutaneous and mucosal tropism [2,4]. *C. lusitaniae,* by contrast, remains a rare opportunistic species, initially isolated from environmental sources and animal intestinal flora, but whose human pathogenicity has progressively gained recognition over recent decades [5]. The distinctive feature of *C. lusitaniae* lies in its dual biological identity: a non-filamentous yeast and possible commensal of mucous membranes, but also a potentially multidrug-resistant opportunistic pathogen, notably against amphotericin B [7]. Its profile distinguishes it from other non-albicans yeasts by its capacity to develop rapid adaptive resistance under therapeutic pressure, a phenomenon attributed to mutations in the ergosterol biosynthesis pathway (ERG3, ERG6) [8].

The cases included in Table 2 were selected from published case reports and case series describing human infections caused by *C. lusitaniae*. Only reports providing sufficient clinical and microbiological information to allow comparison with the present case were included. Epidemiologically, the majority of reported infections due to *C. lusitaniae* concern immunocompromised patients or those hospitalised in intensive care units, often with indwelling medical devices. However, several recent reports describe localised infections in immunocompetent subjects, including pulmonary, cutaneous, and urinary presentations [4]. Our case aligns with this emerging trend, illustrating the possibility of a localised infection due to *C. lusitaniae* in a patient with a generally preserved immune status but with minor metabolic disorders. From a diagnostic standpoint, accurate identification of *C. lusitaniae* remains a mycological challenge. Traditional methods (morphology, subculture on chromogenic agar, carbohydrate assimilation tests) frequently lead to its misidentification as other non-albicans yeasts. The use of MALDI-TOF mass spectrometry or molecular testing now represents the reference standard for reliable identification. The high score obtained in our case attests to the performance of this technology, already recognised as the tool of choice for the rapid detection of rare *Candida *species [6].

**Table 2:** Previously published cases of infections related to Candida lusitaniae. M: male; F: female; y: years; AmpB: amphotericin B

Age	Sex	Infection site	Patient background	Treatment	Outcome	Country	Reference
54 y	M	Pulmonary empyema	Immunocompetent	IV then oral fluconazole	Recovery	USA	Haseeb ul Rasool et al. [4]
15 y	M	Post-lung transplant candidaemia	Immunocompromised	Echinocandin IV	Recovery	Greece	Apsemidou et al. [5]
64 y	F	Candiduria	Diabetic	Fluconazole	Recovery	Morocco	Dahraoui et al. [8]
56 y	M	Candiduria	Diabetic	Fluconazole	Recovery	Morocco	Dahraoui et al. [8]
50 y	M	Candidaemia	Immunocompromised	Micafungin (rapid cross-resistance)	Death (unspecified)	USA	Scott et al. [9]
37 y	F	Fungal stomatitis	Diabetic	Miconazole	Recovery	Italy	Veraldi et al. [10]
55 y	F	Recurrent vaginitis	Immunocompetent	Intravaginal boric acid	Recovery	USA	Silverman et al. [11]
3 y	F	Candidaemia	Immunocompromised	AmB → Caspofungin → Azoles	Death	Switzerland	Asner et al. [12]
46 y	F	Intraperitoneal infection	Immunocompetent	Fluconazole	Recovery	Poland	Wawrysiuk et al. [13]

From a pathophysiological perspective, this yeast may act as a local opportunistic pathogen, particularly when environmental factors create conditions conducive to overgrowth (chronic humidity, microbiome imbalance, local microtrauma, or tympanic scarring). The chronic hypoalbuminaemia observed in our patient may have contributed to a slight impairment of local immune defences, thereby facilitating auricular colonisation. More broadly, the pathogenicity of* C. lusitaniae *is thought to be multifactorial, resulting from a combination of moderate adhesion capacity, variable biofilm formation, cell-surface hydrophobicity, limited enzymatic secretion, and stress-adaptation and morphogenesis-related signalling pathways [14]. Taken together, these virulence traits suggest that *C. lusitaniae* possesses a real, though comparatively modest, pathogenic potential, which becomes clinically significant mainly in immunocompromised hosts.

The therapeutic approach is guided by the site and severity of the infection. For superficial forms, topical polyenes such as nystatin remain effective. Conversely, invasive forms require individualised systemic treatment, often based on second-generation triazoles (voriconazole, posaconazole). *C. lusitaniae *is notable for its intrinsic resistance to amphotericin B and its capacity to develop resistance to flucytosine and azoles [14], with cross-resistance between echinocandins and azoles reported in 30-40% of echinocandin-resistant isolates in the United States [14], as well as the possibility of dynamic heteroresistance induced by fluconazole exposure [7,14]. The choice of topical nystatin in our patient warrants discussion given the presence of tympanic membrane perforation. In our department's clinical experience, nystatin has been used safely in several patients with perforated tympanic membranes, with good tolerance and no observed hearing complications. This is further supported by studies applying nystatin directly to the middle ear, which have shown no evidence of ototoxicity or cochlear hair-cell damage [15]. This is consistent with the favourable outcome observed in the present case, with complete resolution of symptoms and no adverse effects at follow-up. To our knowledge, this represents the first case of otomycosis due to *C. lusitaniae* identified in our department. It also appears to be one of the first published auricular isolates of *C. lusitaniae* in the literature. The favourable outcome of our case following topical nystatin therapy nonetheless suggests that the species retains good clinical susceptibility in superficial infections in immunocompetent hosts.

Finally, the rarity of documented auricular cases of *C. lusitaniae* calls for heightened clinical vigilance. This is particularly relevant as the incidence of non*-*albicans *Candida* yeasts continues to increase, making accurate species-level identification using modern diagnostic techniques essential for guiding appropriate treatment and anticipating potential antifungal resistance.

## Conclusions

This case demonstrates that *C. lusitaniae*, although rarely implicated in otomycosis, can cause localised auricular infection even in the absence of immunosuppression. The phenotypic overlap between* C. lusitaniae* and other non-albicans *Candida *species underscores the importance of accurate fungal identification, which enabled confident species-level diagnosis in this case. Clinical resolution was achieved with topical nystatin alone, with complete resolution of symptoms and no recurrence at one-month follow-up, illustrating a favourable outcome despite the species' recognised potential for acquired antifungal resistance, particularly to amphotericin B, in systemic disease.
